# Martian‑Regolith Simulant Confined Nanogenerators for Wireless Tactile Sensing for Human‐Machine Interface

**DOI:** 10.1002/smll.74734

**Published:** 2026-08-03

**Authors:** Shidhin Mappoli, Keval K. Sonigara, Martin Pumera

**Affiliations:** ^1^ Future Energy and Innovation Laboratory Central European Institute of Technology, Brno University of Technology Brno Czech Republic; ^2^ Office of Research and Development Asia University Taichung Taiwan; ^3^ Faculty of Electrical Engineering and Computer Science VSB‐Technical University of Ostrava Ostrava Czech Republic; ^4^ Department of Medical Research China Medical University Hospital China Medical University Taichung Taiwan

**Keywords:** In situ resource utilization, Martian Regolith, space exploration, tactile sensing, triboelectric nanogenerator

## Abstract

Achieving a sustainable energy system for space missions remains challenging due to the continued reliance on Earth‐supplied materials. This underscores the importance of in situ resource utilization (ISRU) strategies that convert planetary resources into functional electronic components. In this work, we harness the dielectric characteristics of Martian regolith (MR) simulant to create an MR/polydimethylsiloxane (PDMS) composite film with enhanced triboelectric properties. Structural and morphological analyses of the MR reveal multiple oxide‐rich phases, which improve both the dielectric properties and the surface microstructure of the MR/PDMS composite film. The resultant MR/PDMS composite film‐based triboelectric nanogenerator (TENG) delivers an approximately two‐fold increase in open‐circuit voltage compared to the pristine PDMS‐based TENG. The real‐world use of the MR/PDMS TENG is further demonstrated by proof‐of‐concept applications: a glove‐mounted tactile surface sensor with wireless signal transmission and a wearable triboelectric keypad. This work not only showcases advances in MR‐based TENG performance but also marks the first demonstration of triboelectric applications using MR simulants as functional triboelectric material. Additionally, we have demonstrated foundational work toward ISRU‐oriented tactile interfaces incorporating MR‐simulant‐derived functional materials for future controlled habitats and robotic platforms relevant to future space exploration.

## Graphical abstract

A Martian regolith‐simulant/PDMS triboelectric nanogenerator is developed as an ISRU‐inspired, self‐powered tactile sensor for wireless human–machine interface applications.

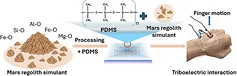

## 1 Introduction

Sustainable power generation is one of the key challenges for long‐duration planetary missions, where dependence on terrestrial resources and bulky energy storage systems limits their operation [1, 2, 3]. Current ISRU strategies are mainly focused on structural applications of extraterrestrial regolith, such as construction materials and radiation shielding, while its potential as a functional material remains unexplored [4, 5, 6, 7]. This is particularly notable in human‐machine interactive tactile systems such as wearable sensors, electronic skins (E‐skins), and interactive robotic interfaces. Space missions traditionally rely on devices such as solar cells and radioisotope thermoelectric generators (RTG). However, in space conditions due to the regular sandstorms and dust deposition, solar cells usually suffer from efficiency losses, while RTGs are reliable and stable, they always carry a risk of nuclear contamination and safety concerns. All this shows the need for lightweight, low‐risk, self‐sufficient power sources.

TENGs offer a scalable solution for harvesting ambient mechanical energy such as vibration, touch, or motion. This mechanical energy‐harvesting property of TENG is used in self‐powered sensing applications across various fields, such as human‐machine interfaces and structural health monitoring [8, 9]. Conventional TENGs rely on the triboelectric series for material selection and pairing [10, 11, 12]. However, such selections are mostly based on synthetic polymers (polytetrafluoroethylene, polyvinylidene fluoride, PDMS, etc.) and metals (aluminum (Al), copper (Cu), etc.), overlooking naturally abundant dielectrics such as MR [13, 14]. Recent advances in TENGs using hybrid polymers, bio‐derived fillers, and nanocomposites have demonstrated improvements in electrical output and mechanical properties [15, 16, 17, 18, 19, 20, 21]. Recent studies have investigated the operational feasibility of TENGs under vacuum and low‐temperature conditions to validate their potential for use in space environments [22, 23, 24, 25]. However, it is worth noting that not all electronic systems on spacecraft are exposed to an extreme space environment. For example, inside the International Space Station, most instruments operate in Earth‐like ambient environments maintained by rigorous Environmental Control and Life Support Systems that regulate the temperature, pressure, and air composition to Earth‐like standards [26, 27, 28]. Most studies on the development of electronic systems focus on terrestrial materials [29]. This shows a critical gap in the use of naturally available dielectrics and abundant mineral resources on planetary surfaces.

MR simulants such as Mars Global Simulant (MGS)‐1, Johnson Space Center Mars‐1A, Jining Martian Soil Simulant‐1, and Yellowknife (Y)‐Mars contain minerals such as hematite, olivine, pyroxene, and plagioclase [4, 30, 31, 32, 33]. These minerals possess distinct dielectric and semiconducting properties. Among these, MGS‑1 was specifically engineered based on Curiosity's Rocknest soil analyses to mimic the mineral composition of MR. These regolith minerals are known to withstand extreme thermal and radiation environments while maintaining compositional stability. Considering this, various regolith materials have already been studied for various structural ISRU applications such as cementation, radiation shielding, and 3D‐printed habitats [6, 34]. Their intrinsic electron affinity and diverse surface chemistry due to the composite material composition also present an opportunity to use them in triboelectric fabrication. Oxide fillers such as BaTiO_3_, TiO_2_, and Fe_2_O_3_ have previously been introduced in polymer matrix to enhance triboelectric layer permittivity and charge‐trapping properties, but such approaches primarily rely on synthetically engineered nanostructures [35, 36, 37, 38, 39]. While MR offers a naturally multiphase mineral system where heterogeneous interfaces can synergistically contribute to interfacial polarization and modification of dielectric response. However, to the best of our knowledge, no prior work has systematically explored triboelectric applications of MR simulants, leaving this combination of planetary materials science and energy harvesting unaddressed.

Here, we report an MR‐PDMS composite‐based TENG (MR‐TENG), in which MGS‐1 MR simulant particles are embedded in a flexible PDMS matrix. This modified composite achieves enhanced dielectric constant, interfacial charge trapping, and tailored surface microstructure, collectively yielding a two‐fold increase in voltage output over pristine PDMS‐based TENG (PDMS‐TENG). Through comprehensive morphological, dielectric, and electrical analysis, we elucidate the mechanisms of performance enhancement. Importantly, the MR‐TENG is evaluated as a mechanical energy‐harvesting device based on its voltage, current, power, and rectified output characteristics. Finally, we demonstrate multiple applications of this MR‐TENG, including a wearable triboelectric keypad and a TENG‐based wireless glove‐mounted tactile surface sensor, validating its feasibility as an ISRU‐compatible energy solution for planetary robotics and wearable electronics.

## 2 Results and Discussion

Figure 1 introduces the overall concept of incorporating an MR simulant as a functional dielectric filler in a PDMS‐based TENG for tactile‐interface applications. Figure 1a shows the fabrication route of MR‐TENG and its application as a wearable touch‐activated sensing system. MR is rich in oxides and silicates such as hematite, olivine, and pyroxene [32, 40]. Considering this, MGS‐1 was selected as a triboelectric filler owing to its inherent dielectric properties and natural stability in extreme conditions [32]. In order to fabricate the negative triboelectric layer, MGS‐1 particles were mixed with PDMS, forming an MR‐PDMS composite (Further details are provided in the supplementary information), which is then deposited onto an Al foil current collector. Multiple such films are then arranged in a 4 × 4 sensor array to function as a self‐powered wearable touch sensor keypad. This platform allows self‐powered signal generation from tactile inputs, enabling real‐time interaction between human operators (users) and robotic/digital systems. Figure 1b illustrates the smart TENG glove useful to astronauts, where tactile gestures from the touch materials of the astronaut suit trigger signal transduction and wireless communication to a space control station/computer. This reduces the dependence on heavy batteries or wired connections and also enables independent communication that can operate in remote and resource‐scarce extraterrestrial settings.

**FIGURE 1:**
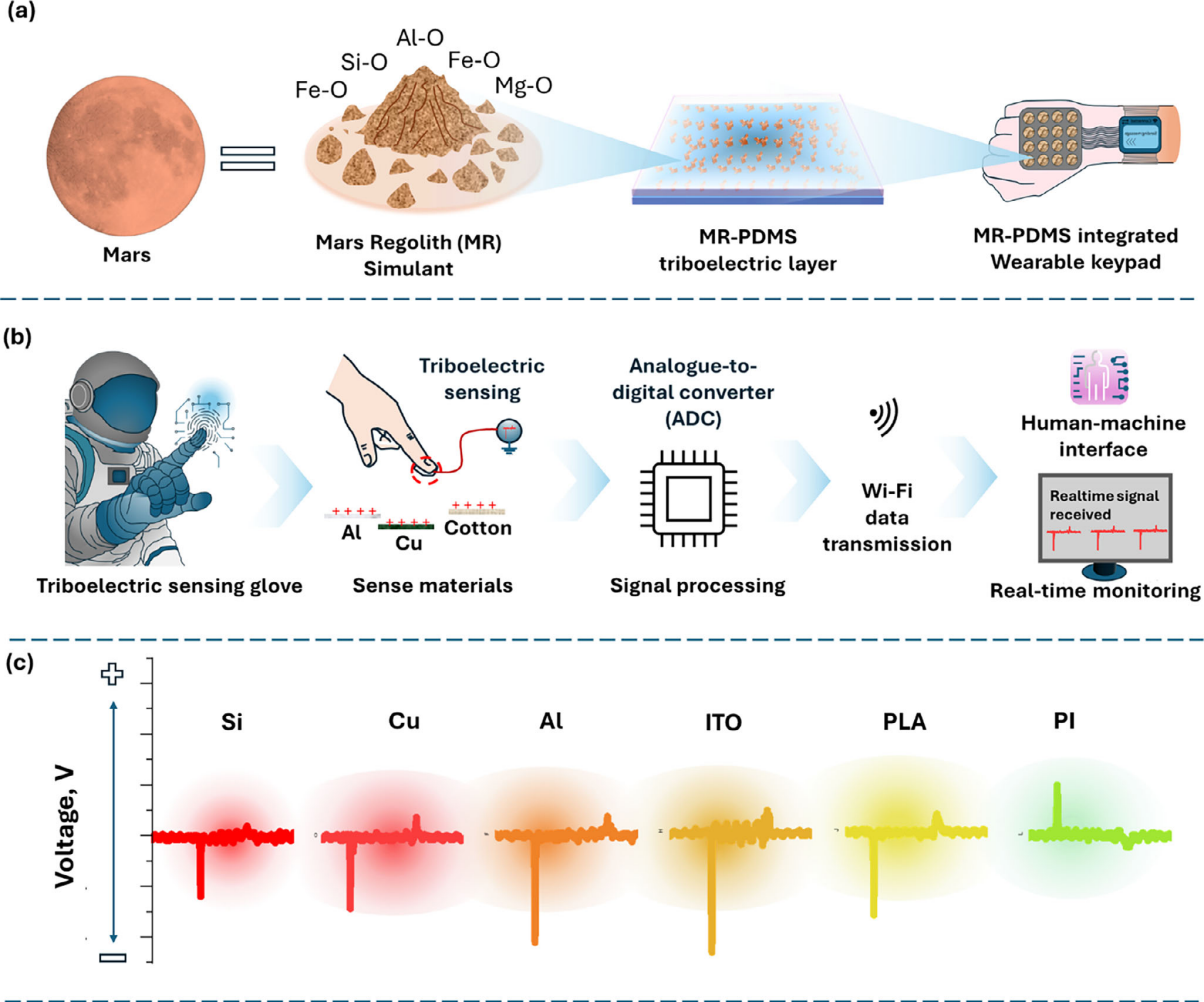
Conceptual overview and triboelectric response of the MR‐TENG. (a) Schematic illustration of the fabrication and operation of MR‐TENG as a wearable tactile keypad. (b) Schematic illustration of a TENG‐based wireless glove‐mounted tactile surface sensor converting touch input to an electrical signal and wireless transmission to a computer. (c) Triboelectric voltage response generated by the MR‐TENG when interfaced with different touch surfaces (Si, Cu, Al, ITO, PLA, and PI).

Additionally, TENGs can be effectively used as a material identification system by studying the triboelectric output voltage patterns. To evaluate the voltage selectivity and responsiveness of the TENG with different materials, MR‐TENG was set up as a wearable tactile probe to study commonly used material surfaces such as silicon (Si), Cu, Al, indium tin oxide (ITO), polylactic acid (PLA), and polyimide (PI). The resulting output voltages (Figure 1c) show the material‐dependent voltage signatures of MR‐TENG, demonstrating its sensitivity and potential for surface classification/detection. The voltage response polarity and amplitude for different material surfaces are also consistent with their relative positions in the triboelectric series. With respect to MR–PDMS, PI generated a positive voltage response, showing relative electron‐accepting behavior, whereas Si produced a net negative response, consistent with relative electron‐donating behavior under the fixed measurement configuration.

### 2.1 Material Characterization

Raman spectroscopy of the MR simulant was performed to understand the structural and chemical composition, as given in Figure 2a. The MR simulant shows multiple vibrational fingerprints that show a multicomponent basaltic origin. In low‑frequency region of the Raman spectra is dominated by the hematite multiplet with A1g mode at ∼224 cm^−1^, followed by Eg bending at ∼299 cm^−1^ and ∼430 cm^−1^, and a symmetric Fe–O stretch at ∼605 cm^−1^ [41, 42, 43, 44]. In the mid‑frequency region, two doublets at ∼822 cm^−1^ and ∼855 cm^−1^ arise from the ν1 (Si‐O symmetric) and ν3 (Si‐O asymmetric) stretching modes of (Mg, Fe)_2_SiO_4_ olivine [45, 46]. Higher wavenumber bands at ∼918 cm^−1^ and ∼962 cm^−1^ denote the traces of low‑Ca (enstatite) and high‑Ca (augite) pyroxenes. The peak centered at ∼1522 cm^−1^ is attributed to two‑phonon scattering in hematite and minor amorphous silicate content [43, 47, 48]. This supports the MGS‐1 simulant's mineralogical similarity to Martian basalts. These phases explain the dielectric enhancement and the significant increase in open‑circuit voltage achieved due to the MR loading, connecting Raman vibrational fingerprints to triboelectric performance.

**FIGURE 2:**
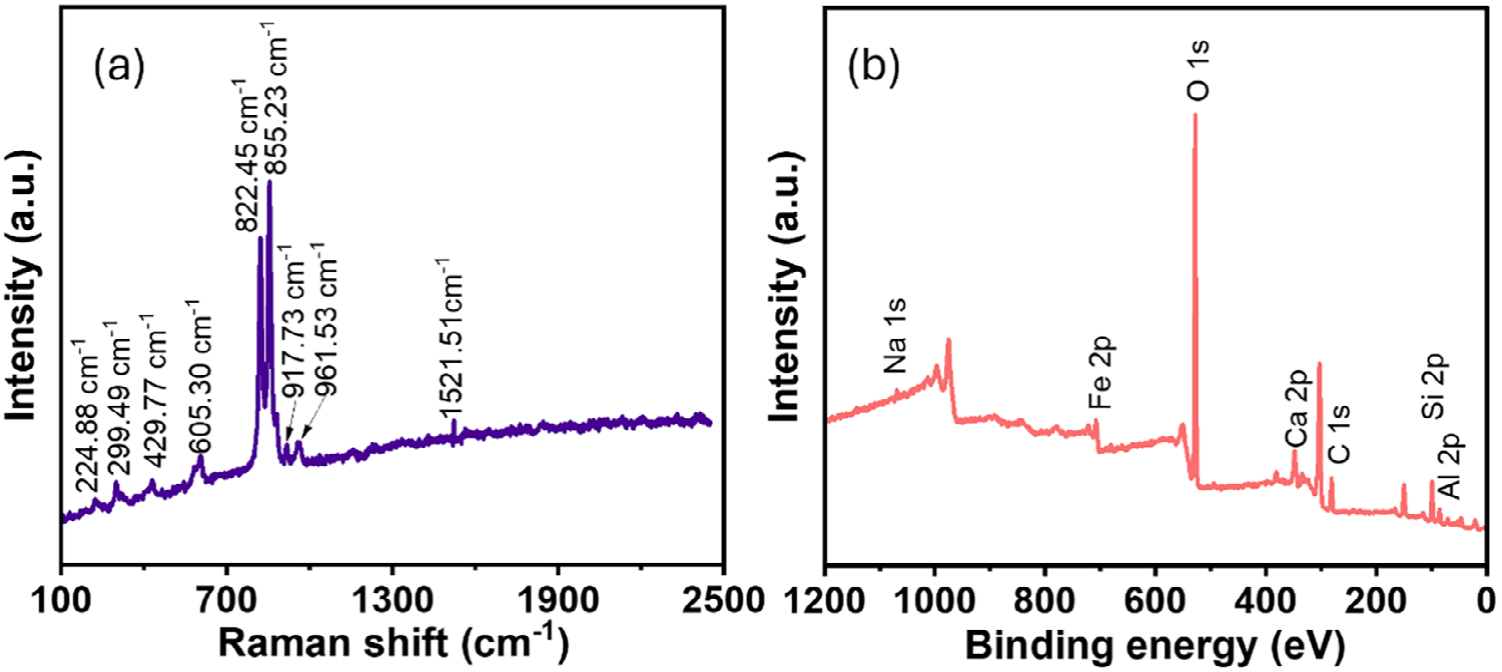
Structural and compositional characterization of MR. (a) Raman spectrum showing characteristic vibrational modes of mineral phases. (b) x‐ray photoelectron spectroscopy spectrum of pure MR.

X‐ray diffraction (XRD) analysis was carried out to understand the crystalline phases present in the MR simulant. The XRD diffractogram (Figure S1) demonstrated sharp Bragg reflection patterns, confirming the multiphase crystalline nature of the material. The mineralogical composition observed was consistent with that specified by the manufacturer. The XRD peaks consist primarily of plagioclase, pyroxenes, olivine, epsomite, ferrihydrite, hydrated silica, and iron oxides. Additionally, with an amorphous contribution that can be associated with basaltic glass [33, 48, 49]. The XRD results confirm that the MR simulant mineralogically resembles the basaltic minerals reported for Martian planetary surface materials.

Furthermore, x‐ray photoelectron spectroscopy (XPS) analysis of the MR was performed to investigate the surface chemistry (Figure 2b). The survey spectra show O 1s and Si 2p major peaks, which can be attributed to silicate frameworks. Furthermore, the Al 2p peak validates the plagioclase presence, and the Fe 2p doublet shows the Fe(III/II) oxides and Fe‐containing silicates (hematite/magnetite/pyroxenes/olivine). Besides, traces of Mg 1s, Na 1s, and Ca 2p were also observed from survey spectra. The survey spectrum also shows a C 1s peak, which could be due to intrinsic carbonate phases and carbon on the MR surface. These observations confirm that the surface composition of the simulant is consistent with its reported bulk mineralogy [32, 50].

Following characterization of the pristine MGS‐1 particles, their incorporation into PDMS was investigated across composite films containing 5, 10, and 15 wt% MR filler, with pristine PDMS used as a reference for subsequent comparison of dielectric and triboelectric performance. The scanning electron microscopy (SEM) image of MR and an MR‐PDMS composite was observed to understand the surface morphology and microstructures of the sample. SEM imaging reveals that the simulant consists of a highly irregularly shaped powder structure (Figure 3a). After dispersion in PDMS (Figure 3b), individual grains remain well anchored with some protruding above the polymer surface. This creates a surface roughness that is absent in pure PDMS films. EDX elemental maps (Figure 3c–j) reveal a chemically heterogeneous elemental distribution. Overall, MR was dominant with Si and oxygen, outlining the silicate framework consistent with olivine and pyroxene assignments previously observed from Raman and XRD peaks. Quantitative EDX elemental mapping analysis yields O 68.3 at%, Si 13.3 at%, Al 6.1 at%, Fe 3.8 at%, Mg 3.7 at %, Ca 2.2 at %, S 1.2 at %, Na 1.2 at%, with trace amounts of K and Cu (Figure S2). These proportions are consistent with the reported mineralogy of the MGS‐1 simulant [32, 50]. Furthermore, EDX mapping of the MR–PDMS composites at increasing loadings (5, 10, and 15 wt%) revealed a systematic increase in elemental at% of the MR phases. In particular, Fe, Al, and Cu intensities increased progressively with an increase in filler concentration (Figure S3).

**FIGURE 3:**
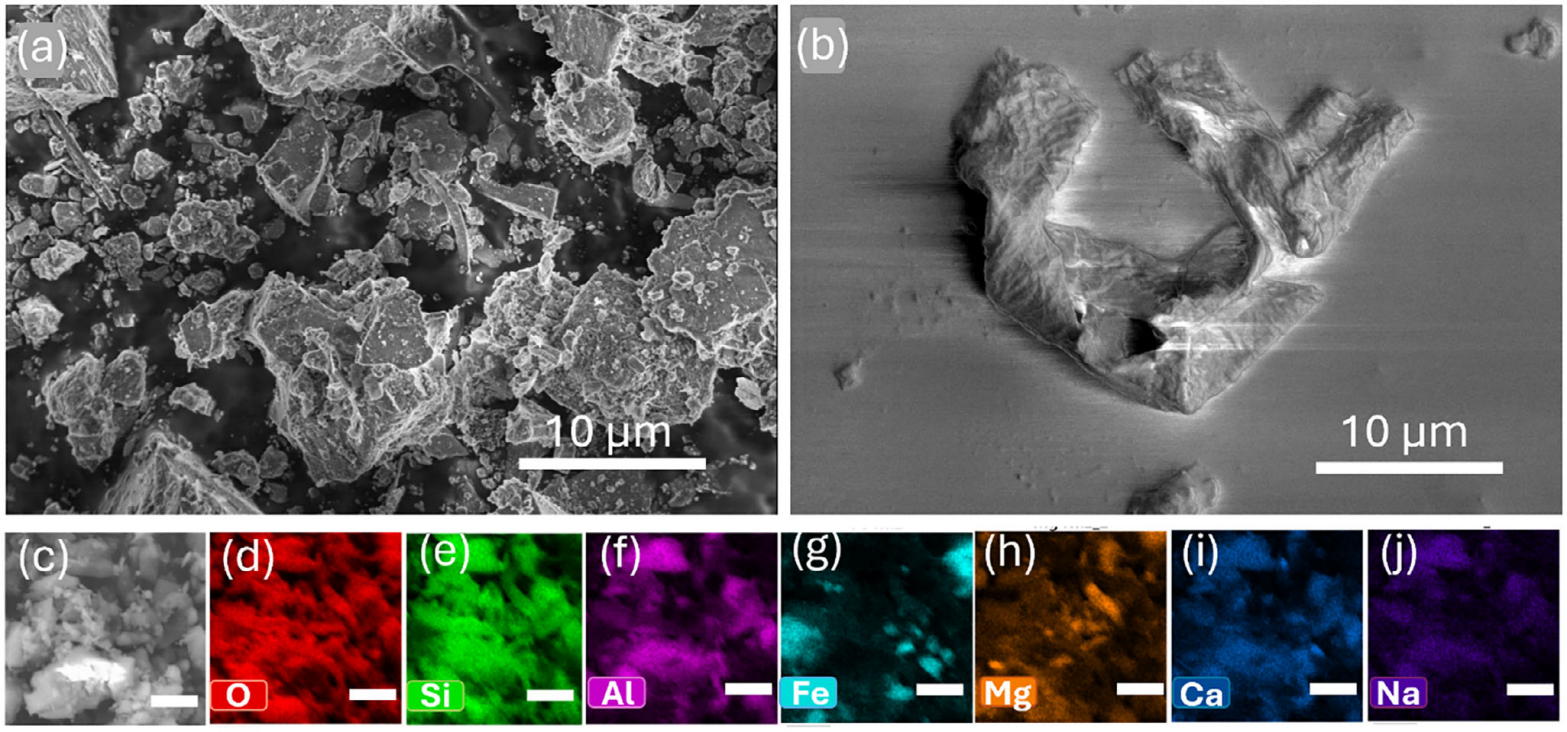
Morphological and elemental characterization of MR. Scanning electron microscopy image of (a) MR particles showing their surface morphology, (b) MR embedded in a PDMS matrix. (c–j) EDX elemental mapping of the MR particles, highlighting the spatial distribution of key elements. Scale bar: 5 µm.

Confocal laser scanning microscopy (CLSM) was employed to further understand the morphological changes with an increase in MR loading in PDMS. Surface intensity micrographs (Figure 4a–d) and their corresponding false‐color topographical maps (Figure 4e–h) reveal a progressive increase in surface roughness as the MR content increases from 0 to 15 wt%. Pristine PDMS (Figure 4a,e) exhibits a smooth surface with a root‐mean‐square roughness (Sq) of 0.95 µm. Upon incorporation of 5 wt% MR (Figure 4b,f), the Sq is increased to 1.15 µm, with a change in surface topology with micro‐asperities while preserving the continuity of the polymer matrix. With 10 wt% loading (Figure 4c,g), Sq further rises to 1.29 µm, with further pronounced protrusions emerging on an otherwise planar surface of PDMS. A large change in morphology can be observed at 15 wt% MR (Figure 4d,h), where the roughness rises to 3.81 µm. These sharp height gradients can induce electric field screening and introduce additional charge leakage pathways, potentially reducing the triboelectric performance.

**FIGURE 4:**
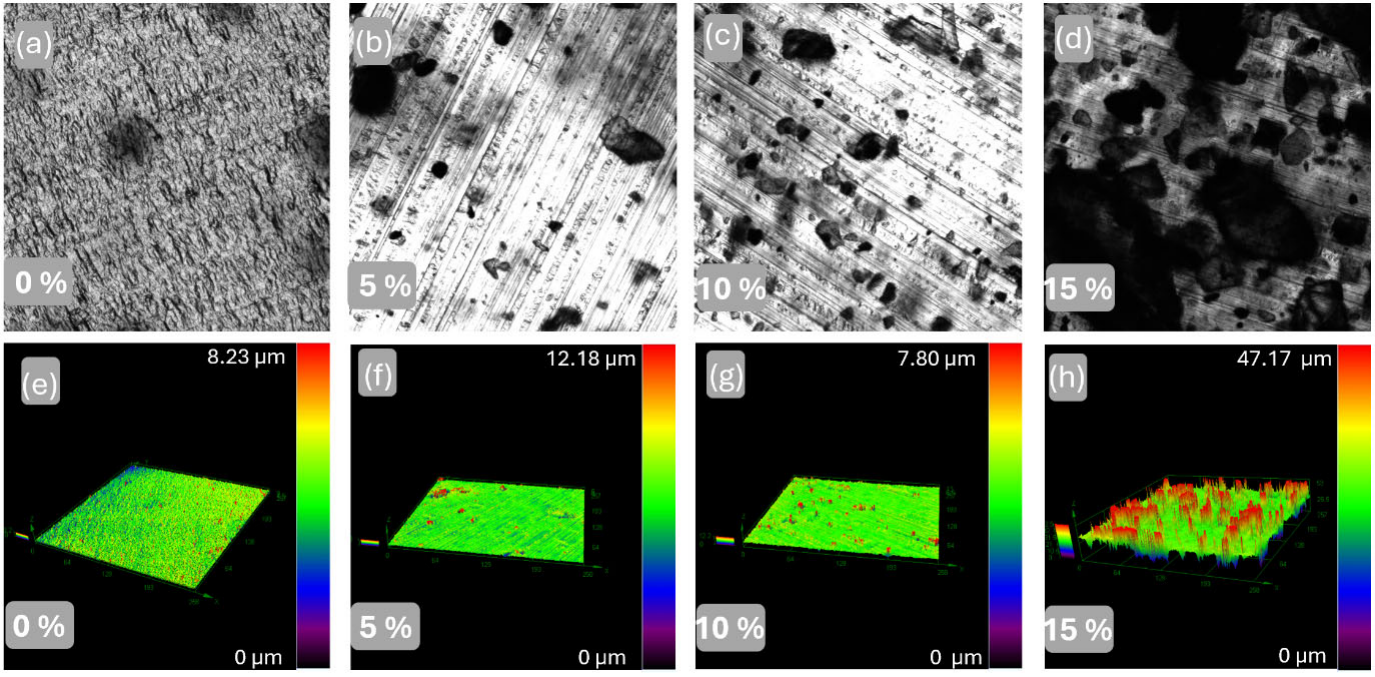
Surface morphology and topography of MR–PDMS composites. (a–d) Optical confocal microscopy intensity maps of PDMS composites containing 0 wt%, 5 wt%, 10 wt%, and 15 wt% MR, respectively. (e–h) Corresponding 3D false‐color topographical maps derived from CLSM analysis. The lateral scan size for all images is 258 × 258 µm^2^.

### 2.2 Working Mechanism of the MR–PDMS TENG

Figure 5 illustrates the working mechanism of the MR‐PDMS‐based TENG. The MR‐TENG operates in classic contact‐separation mode, which is fundamental to TENG charge generation [51]. (a) Contact state (pressed): When the MR‐PDMS electrode and Al electrode are in contact, electrons transfer from the Al electrode to the MR‐PDMS electrode surface due to differences in their electron affinity and work function. The presence of hematite‐rich MR enhances electron affinity at the MR‐PDMS surface [52]. This will enhance the charge transfer and electron trapping in the interfacial region. The charge density equation is given by:

$$\sigma =\frac{Q}{A}$$

where σ denotes surface charge density, Q is the total charge transferred, and A is the area of contact. The increase in surface roughness and dielectric constant due to the MR grains will contribute to the increase *Q* and localized electric field strength. (b) Separation (Releasing): When mechanical pressure is released, the MR‐PDMS electrode will start to separate and move away from the aluminum electrode (non‐contact). This separation between electrodes results in the decrease of interfacial capacitance (C with increasing gap d. This effectively results in an increased potential difference.

$$C=\frac{\varepsilon A}{d}$$

**FIGURE 5:**
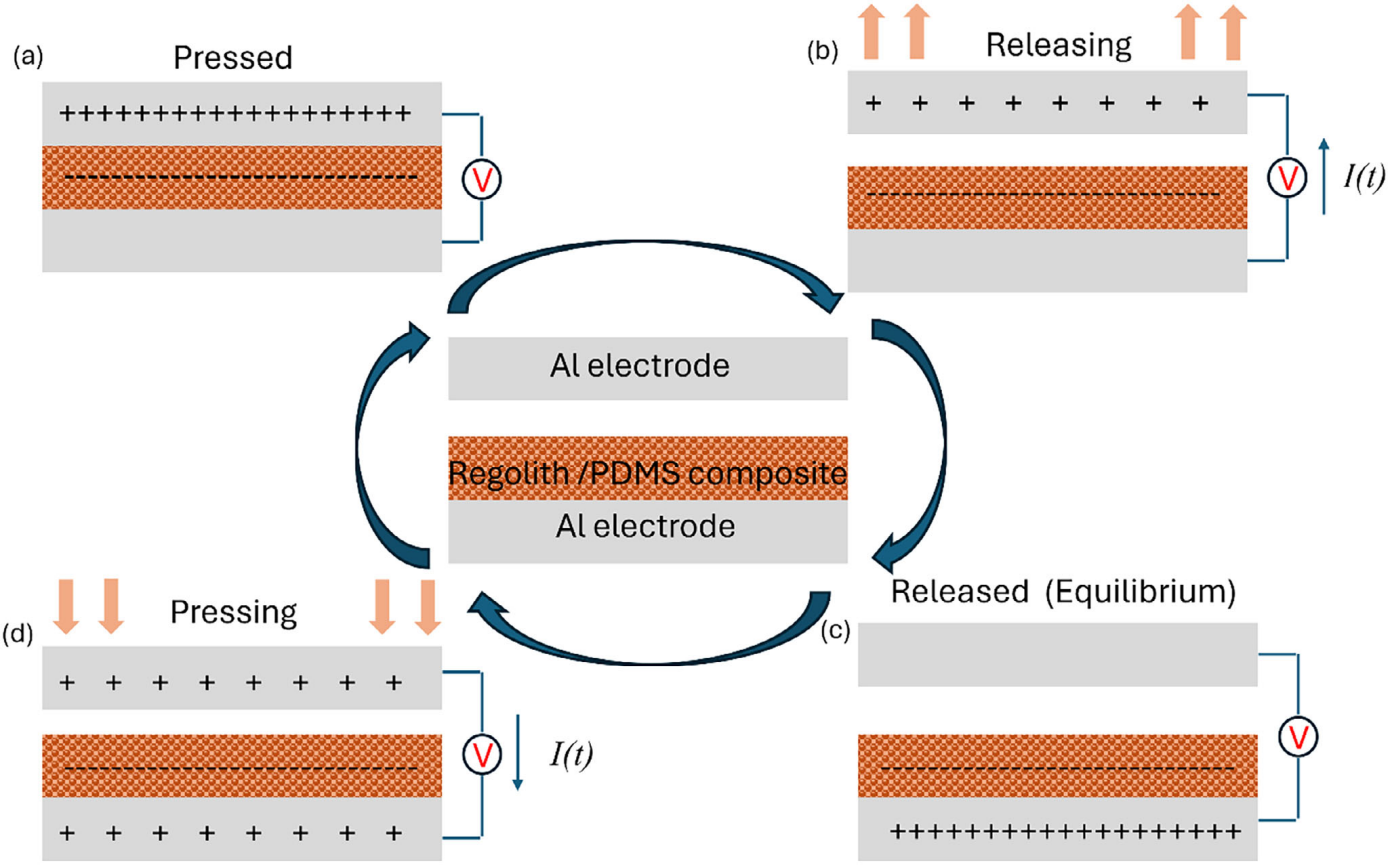
Working mechanism of the MR‐TENG. Schematic illustration of the triboelectric charge generation during contact‐separation cycles: (a) Contact state during pressing; (b) separation initiated during release; (c) fully released state showing charge transfer; (d) re‐contact during subsequent pressing cycle.

Additionally, the MR grains in the PDMS matrix will also create a localized electric field, promoting efficient separation and accumulation of triboelectric charges.

$$V=\frac{Q}{C}$$

This results in an increase in voltage in the external circuit [53]. The MR grains (particularly hematite and pyroxene crystals) facilitate efficient dielectric polarization and charge retention. (c) Fully Released State: At the maximum separation stage, the C will reach the lowest value and thereby maximize the potential difference (V *)*. This results in electrons flowing through the external circuit due to the high potential difference, generating a positive half‐cycle voltage output from the MR‐TENG. (d) Re‐contact (Pressing): As the materials are pressed again, the gap between the electrodes closes, and this increases the interfacial capacitance. This causes charges to flow in the opposite direction, producing a negative half‐cycle in the MR‐TENG output voltage.

### 2.3 Electrical Performance Analysis

The quantitative evaluation of electrical output performance was performed on PDMS‐TENG (Pure PDMS) and MR‐TENG (MR/PDMS composite) by measuring the variation of AC (alternating current) open‐circuit voltage ($V_{\mathrm{oc}}$) and short‐circuit current ($I_{\mathrm{sc}}$) at different frequencies of 6, 8, 10, and 12 Hz. This provides an insight into the influence of MR content on the MR‐TENG output (Figure 6). The PDMS‐TENG with an Al counter electrode generates a $V_{\mathrm{oc}}$ of approximately 6.1 V (peak, $V_{\mathrm{pp}}$ 8.3 V) (Figure 6a) and an $I_{\mathrm{sc}}$ of approximately 5.0 µA (peak, $I_{\mathrm{pp}}$ ≈ 8.4 µA) (Figure 6b) at 12 Hz. With the increase in tapping frequency from 6 to 12 Hz with a contact force of 2.8 N, a notable enhancement in electrical output ($V_{\mathrm{oc}}$, $I_{\mathrm{sc}}$) is observed. This frequency‐dependent enhancement in measured electrical output may be associated with the increased movement speed of the triboelectric layer and the resulting higher charge‐transfer and external electron‐flow rates at increased working frequency, consistent with previous reports on contact‐mode and tactile TENG systems [13, 14, 21, 54].

**FIGURE 6:**
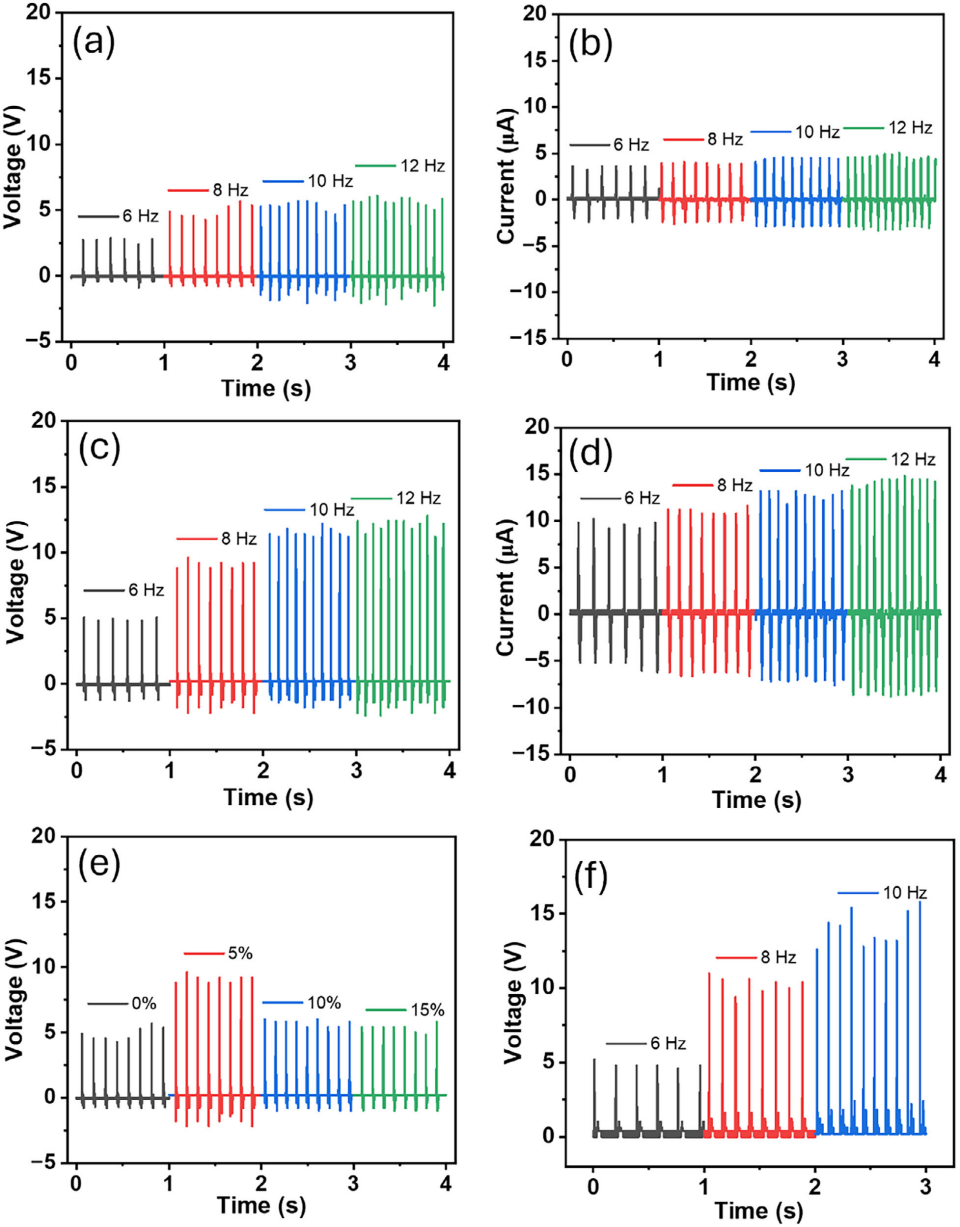
Electrical performance of MR–PDMS‐based TENGs. (a) Variation of AC open‐circuit voltage ($V_{\mathrm{oc}}$) with frequency for PDMS‐TENG. (b) Corresponding Short circuit current ($I_{\mathrm{sc}}$) output of PDMS‐TENG. (c) $V_{\mathrm{oc}}$ of 5 wt% MR‐TENG. (d) $I_{\mathrm{sc}}$ of 5 wt% MR‐TENG. (e) Comparative voltage output of TENGs with varying wt% MR concentrations (0%, 5%, 10%, and 15%). (f) Rectified voltage output of the MR–PDMS TENG.

When 5 wt% MR is added to PDMS, it enhances the TENG output performance of the device in the measured frequencies. The $V_{\mathrm{oc}}$ was enhanced over 2 times, reaching 12.8 V (peak, $V_{\mathrm{pp}}$ ≈ 15.2 V) at 12 Hz (Figure 6c). Similarly, the $I_{\mathrm{sc}}$ also increases proportionally to about 14.8 µA (peak, $I_{\mathrm{pp}}$ ≈ 23.6 µA) at 12 Hz (Figure 6d). A similar frequency‐dependent increase in output voltage is also observed in MR‐TENG, but with a higher output voltage than in PDMS‐TENG under a contact force of 2.8 N. These enhancements in electrical output can be attributed to the synergistic interplay of mechanisms: (i) the improved dielectric properties due to the MR particles enhance charge storage; (ii) MR grains increase effective contact area and local field concentration due to surface micro‐roughness; and (iii) the oxide‐rich MR composition provides abundant electron‐trapping sites that stabilize surface charges and reduce recombination losses. Collectively, these attributes improve the charge density and triboelectric conversion efficiency. This underscores the role of MR incorporation in modulating TENG performance. Consequently, the MR‐TENG generates a power density of 5.9 µW cm^−2^ and an energy density of 0.494 µJ cm^−2^ cycle^−1^ at 12 Hz. While engineered oxide fillers such as BaTiO_3_ and TiO_2_ have been extensively investigated for dielectric enhancement in TENG systems due to their high permittivity and strong interfacial polarization capability [38, 39, 55, 56]. It should be noted that the MR‐based composite presented in this work is not intended to compete with engineered oxide/ferroelectric fillers in terms of absolute dielectric constant, but rather to study a naturally abundant, multifunctional, and ISRU‐compatible alternative for extraterrestrial triboelectric energy harvesting applications. The heterogeneous mineral composition of MR enables interfacial polarization and charge trapping while avoiding the need for complex synthesis or surface functionalization procedures commonly associated with engineered nanofillers, also maintaining material simplicity and environmental relevance.

To evaluate the influence of MR loading and assess the optimal concentration, voltage comparison was conducted at higher filler contents (0, 5, 10, and 15 wt%) (Figure 6e). This shows a nonlinear dependence of output performance on filler loading, with 5 wt% MR loading demonstrating the highest voltage performance. With further increases in MR concentration to 10% and 15%, a decrease in $V_{\mathrm{oc}}$ was observed. This reduction in $V_{\mathrm{oc}}$ after a threshold concentration can be due to particle agglomeration and formation of surface asperities observed previously with CLSM mapping, which generate localized field screening and charge leakage. This could disrupt effective interfacial charge separation and suppress triboelectric performance. For practical applications, the AC output from TENGs must be rectified into DC to connect with electronic systems. To demonstrate this, the MR‐TENG output was connected to a full‐wave rectifier. The resulting rectified $V_{\mathrm{oc}}$ at different frequencies was observed (Figure 6f). This demonstrates the feasibility of translating the AC pulses of the MR‐TENG into a usable power supply, thereby bridging between mechanical energy harvesting and real‐world electronic applications.

To further explore the possibility of using a different counter electrode, additional measurements were carried out using cotton fabric as the tribopositive electrode. Figure S4 shows the electrical output profiles of the pure PDMS (Figure S4a,b) and 5 wt% MR–PDMS composite (Figure S4c,d). The MR‐TENG yielded open‐circuit voltages of up to 17.0 V peak, ($V_{\mathrm{pp}}$ ≈ 22.0 V) and currents of 14.8 µA (peak), ($I_{\mathrm{pp}}$ ≈ 23.6 µA) at 12 Hz tapping frequency. Importantly, the relative enhancement trend with filler loading was similar (Figure S5) and devices with 5 wt% MR consistently showed improved performance compared to both pristine PDMS and higher‐loading MR‐composites (10–15 wt%). This confirms that the optimal concentration is independent of the counter electrode.

The MGS‐1 simulant has a heterogeneous multiphase mineral composition, and each mineral contributes distinct dielectric and interfacial functionalities within the PDMS matrix. So, the enhanced performance of MR–PDMS TENGs can be attributed to the synergic effect of the multiphase composition of MGS‐1. XRD analysis supports the presence of feldspars, Fe–Mg silicates, and Fe oxides phases in MGS‐1. Additionally, an amorphous fraction containing basaltic glass and hydrated silica constituents has been reported for the MGS‐1 formulation [32]. These heterogeneous mineral constituents may collectively contribute to the effective dielectric response of the MR–PDMS composite. Feldspar‐ and Fe–Mg silicate‐type phases are dielectric minerals whose relative permittivity is generally higher than that of PDMS, and they can contribute to the effective dielectric response and interfacial polarization of the MR–PDMS composite [57]. Minor Fe oxides may also influence the composite dielectric constant and charge‐trapping behavior [58]. Collectively, these heterogeneous minerals raise the effective dielectric constant, the interfacial trap density, and the surface topography.

To gain insight into the effect of MR incorporation on the dielectric properties of PDMS, the frequency‐dependent dielectric properties of MR–PDMS composites with 0, 5, 10, and 15 wt% MR loadings were investigated (Figure S6a). The non‐monotonic dielectric behavior of the MR–PDMS composites may arise from the combined effects of filler distribution, interfacial polarization, and dielectric relaxation within the composite matrix. The relative permittivity (ε′) shows a higher value than that of pure PDMS at a 5 wt% MR concentration across the measured frequency range. This enhancement in ε′ at 5 wt% MR may be associated with the incorporation of inorganic oxide‐ and silicate‐rich mineral phases, which generally possess higher relative permittivity than PDMS and can increase the effective dielectric response of the composite [59]. In heterogeneous polymer–inorganic filler systems, dielectric contrast between the filler and polymer matrix can also promote interfacial charge accumulation, commonly described as Maxwell–Wagner–Sillars‐type interfacial polarization [60]. However, at higher loadings (10 wt% and 15 wt%), a clear drop in ε′ is observed relative to the 5 wt% MR–PDMS composite. This behavior suggests that the effective dielectric response is not governed solely by the intrinsic permittivity of the filler, but also by filler dispersion, filler–matrix interfacial quality, local microstructural heterogeneity, and dielectric relaxation within the composite matrix. Similar non‐monotonic dielectric behavior has been reported in inorganic‐filled silicone‐rubber composite systems [61]. This can be correlated with the drop in the electrical output of 10 wt% and 15 wt% MR‐loaded TENG. Since the MR–PDMS films exhibit non‐uniform surface morphology at higher doping of 10% and 15% and the elastically deformable nature of PDMS, subtle variations in electrode/composite contact and local air gaps at the electrode/composite interface during sandwiching may influence the measured properties.

Figure S6b illustrates the corresponding dielectric loss tangent (tanδ) across the same frequency spectrum (10^−2^ Hz to 10^6^ Hz) of MR–PDMS composites with varying filler loadings, highlighting energy dissipation mechanisms within each composite. Pristine PDMS exhibits high tanδ values in the lower measured frequencies. This indicates the minimal polarization energy dissipation and stable dielectric behavior [62]. The incorporation of MR modifies the dielectric loss response owing to changes in interfacial polarization and relaxation processes at the MR–PDMS interfaces. Notably, the 15 wt% and 10 wt% MR–PDMS composite exhibits the lower tanδ value, particularly in the low‐frequency region under the investigated conditions. This indicates comparatively reduced dielectric energy dissipation at this composition. The 5 wt% composite exhibits a moderate dielectric loss profile and the highest relative permittivity. This indicates an effective balance between enhanced polarization response and limited dielectric dissipation. Therefore, the superior TENG performance of the 5 wt% MR–PDMS composite is attributed primarily to its enhanced effective dielectric polarization and corresponding charge‐storage capability, rather than solely to the lowest dielectric loss.

Furthermore, the long‐term operational stability of the MR–PDMS TENG was carried out under longer cyclic operation (Figure S7). The output voltage was monitored at regular intervals of 0, 3, 6, and 9 h. The device exhibited stable triboelectric performance, with V_pp_ values of 12.1, 11.55, 10.72, and 11.52 V, respectively, retaining 95.2% of its initial output after 9 h. The minor fluctuations observed in the V_pp_ are likely associated with variations in contact conditions and interfacial charge redistribution during repeated contact–separation cycles rather than permanent material degradation. This indicates a good operational durability of the MR–PDMS composite device for long‐term operation.

### 2.4 Applications

Beyond energy harvesting, TENG outputs can be used as interactive sensing platforms. We demonstrate proof‐of‐concept demonstrations of MR‐TENG to illustrate their application: a glove‐mounted tactile surface sensor with wireless signal transmission and a wearable triboelectric keypad. These demonstrations highlight the potential of MR–TENGs to be used as autonomous, ISRU‐compatible components for human–machine interfaces in both terrestrial and extraterrestrial settings.

Surface material identification is essential for autonomous robotic exploration and smart wearables. However, conventional sensors rely on external power sources. The fabricated MR–TENG‐based touch sensor prototype is shown in Figure 7a, while the operation mechanism of the touch‐based material sensor is schematically illustrated in Figure 7b. The MR–PDMS patch (∼1 cm^2^) mounted on a fingertip of the gloves was fabricated. This acts as a self‑powered probe that produces a unique voltage output depending on the surface in contact/touch. This signal structure depends on the triboelectric property of the surface material. The finger tapping response to six representative materials, such as Si wafer, Cu foil, Al sheet, ITO, PLA and PI, was observed and generates distinct corresponding waveforms (Figure 7c). The voltage waveforms show two distinct response types. Electron‐donating surfaces (Si, Cu, Al, ITO, PLA) produce prominent negative signals, with voltage ranging from −0.33 V (Si) to −0.95 V (ITO). While the positive side of the peak remains low (< +0.1 V), confirming a donor‐type voltage polarity. In contrast, the electron‐accepting type surface (PI) exhibits a prominent positive voltage profile, generating pulses between +0.17 and +0.38 V with only minor negative components (∼ −0.1 V). This polarity inversion provides a strong binary criterion for material discrimination.

**FIGURE 7:**
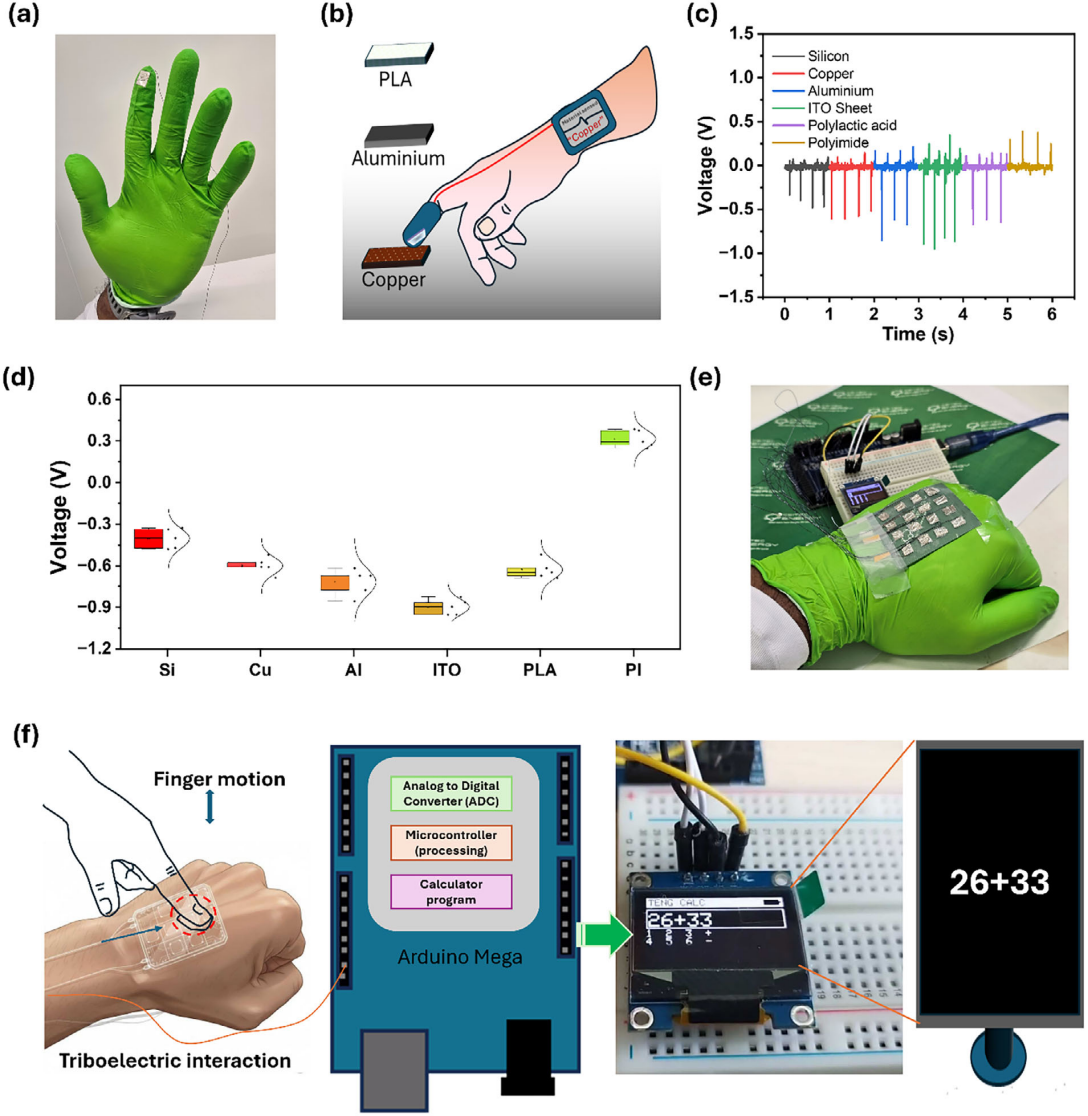
Touch‐based material detection and glove‐mounted keypad application using the MR–TENG. (a) Photograph of a material‐specific touch detection utilizing MR–PDMS composite as the active sensing layer (glove‐mounted tactile surface sensor). (b) Schematic illustration of the triboelectric mechanism for material‐specific touch detection using the MR‐TENG. (c) Voltage response generated by touching the TENG surface with different materials. (d) Statistical distribution (box plots) of peak voltages across multiple taps of glove‐mounted tactile surface sensor. (e) Demonstration of the MR‐PDMS TENG integrated into a flexible glove‐based keypad. (f) Schematic illustration of the circuit model of TENG integrated into a flexible glove‐based keypad for a calculator.

To validate these experimental observations statistically, the peak voltages from multiple taps are illustrated using a box plot (Figure 7d). Donor‐type surfaces (Si, Cu, Al, ITO, PLA) exhibit consistently negative voltage medians. This spans from −0.40 V (Si) to −0.90 V (ITO), while PI remains positive with a median of +0.30 V. The narrow interquartile ranges of the box plot show the reproducibility of the response, with minor fluctuations with tapping. The ITO shows the deepest negative voltage peak, whereas PI maintains a positive voltage distribution showing an evident acceptor signature. The polarity separation and amplitude clustering confirm that surface material identity can be determined under controlled conditions based on characteristic voltage signatures. However, it should be noted that the triboelectric response of the system can be influenced by environmental factors, such as humidity, temperature, and contact conditions, which affect surface charge density and charge transfer processes [63, 64, 65]. Therefore, the material identity determination demonstrated in this work was performed under controlled laboratory conditions to ensure that the observed voltage differences were primarily due to intrinsic material properties, including triboelectric polarity and dielectric characteristics.

To realize a human‐machine interface in remote locations, the sensing devices should be low‐power, durable, and scalable inputs. To convert triboelectric sensing into a practical human‐machine interface, the MR‐PDMS composite was configured into a self‐powered triboelectric keypad. This device consists of a 4 × 4 unit array of MR composite films (1 cm^2^ each), individually laminated onto Al electrodes and separated by an insulating substrate layer (Figure S8). Each key was separately wired to an independent readout channel of the Arduino Mega 2560 analogue input pin, enabling spatially resolved signal acquisition. The individual pads were then mounted onto flexible polyethylene terephthalate sheet, yielding a lightweight, mechanically robust assembly suitable for repetitive tactile input.

When pressed with a fingertip, the corresponding key generates a triboelectric pulse that can be assigned to an individual number, character, or function. The signals were acquired without external bias or amplification, confirming the self‐powered operation of the keypad (Figure 7e). A schematic illustration of the circuit model of TENG integrated into a flexible glove‐based keypad for a calculator is shown in Figure 7f. To demonstrate functionality, the keypad was further integrated with an Arduino‐driven OLED display, enabling real‐time calculator operations to directly function with triboelectric voltage inputs (Video S1). This proof‐of‐concept highlights MR–PDMS TENGs can also function as ISRU‐compatible computing interfaces. Such systems could enable sustainable human–machine interfaces for terrestrial electronics as well as extraterrestrial applications, including rover control panels, habitat user interfaces, and astronaut suit modules. Compared to conventional capacitive or resistive touch panels that demand a continuous power supply and sophisticated detection circuits, this triboelectric keypad offers a power‐autonomous alternative that directly transduces tactile events into voltage pulses.

Wireless signal transmission is critical for extraterrestrial sensing systems in operations that need remote operation. To demonstrate this capability, the MR–PDMS TENG was interfaced with a LilyGO T‐Display module. The real‐time analogue voltage pulses from the TENG, when pressed, were directly fed into the analog‐to‐digital converter (ADC) input pin of the LilyGO module and processed in real time. Using the built‐in Wi‐Fi interface, the acquired data were wirelessly transmitted to a host computer, where corresponding values for voltage were displayed, in addition to the inbuild display. TENG output was directly connected without the need for intermediate amplification circuits (Video S2). Such a wireless self‐powered interface extends the function of triboelectric devices beyond localized sensing to distributed communication networks. In extraterrestrial scenarios, wireless TENG inputs may provide untethered control panels or gesture‐based communication tools for rovers and habitats, where wired systems are less desirable and energy autonomy is crucial.

Collectively, these findings establish MR as more than a passive structural medium. When integrated into a polymer, it can function as an active dielectric component that can be used for various applications such as self‐powered energy harvesting, tactile input, and wireless signal transmission. This convergence of TENGs with ISRU concepts points toward a pathway for sustainable, self‐reliant electronics on Earth and in extraterrestrial environments. It should be noted that the present proof of concept demonstration is targeted toward ambient, enclosed environments expected in extraterrestrial missions rather than direct exposure to harsh and open space conditions.

## 3 Conclusion

This study shows the viability of Martian regolith as a functional dielectric filler for polymer‐based TENGs, offering a significant step toward sustainable, self‐sufficient electronic systems for space exploration. By achieving over two‐fold increase in electrical output over pristine PDMS, the MR–PDMS demonstrates the effectiveness of leveraging local planetary materials. Structural and morphological analysis shows this performance enhancement to the synergic effect of rich mineralogy and optimized surface microstructure of the composite. The successful demonstration of a touch‑based material sensor confirms the practical feasibility of this approach and its potential to transform ISRU strategies for future missions. While further studies are needed to address durability and scalability, these findings pave the initial path for broader adoption of locally sourced electronic solutions in extraterrestrial exploration. The feasibility of MR‐polymer composites as triboelectric media for ISRU‐enabled electronic platforms is studied for operation under controlled and ambient conditions. Future efforts may further extend testing to vacuum, radiation, and thermal extremes to expand applicability to fully exposed space systems.

## 4 Experimental Section

### 4.1 Materials

Polydimethylsiloxane (PDMS, Sylgard 184) Elastomer Kit. The kit contains a silicone elastomer base and curing agent, which was procured from Dow, USA. MR simulant (MGS‐1) was acquired from Space Resource Technologies (USA). The PLA filaments for 3D printing were obtained from Filament2Print, Spain. All chemicals were of analytical grade and used as received.

### 4.2 Preparation of MR‐PDMS Composite Films

The MR simulant was ball‐milled in a stainless‐steel jar at 25 Hz for 20 min using a CryoMill (Retsch) (CRYOMILL) prior to composite preparation to achieve a smaller particle size distribution. PDMS base and curing agent were thoroughly mixed at a 10:1 w/w ratio, followed by the incorporation of ball‐milled MR at loading concentrations of 5, 10, and 15 wt%. The mixtures were stirred mechanically to ensure homogeneous dispersion. Subsequently, the composite mixture was doctor‐bladed into a rectangular film (4 cm × 2 cm) on Al foil and cured at 70°C for 4 h in a hot air oven.

### 4.3 Fabrication of TENG

The cured MR‐PDMS composite film served as the negative triboelectric layer, while Al foil functioned as the positive electrode. The two electrodes were assembled in a vertical contact‐separation configuration with a spacer ensuring consistent separation of 2 mm. The triboelectric layers were mounted on 3D‐printed PLA substrates, which provided mechanical support during contact–separation operation. An electric linear motor actuator with a hub of 20 mm (24 V DC, max 1000 rpm, DAOE) induced periodic mechanical contact and separation at frequencies of 6, 8, 10, and 12 Hz. The applied contact force during operation was maintained constant at 2.8 N for all measurements.

### 4.4 Electrical Characterization

The open‐circuit voltage ($V_{\mathrm{oc}}$) of the TENG devices was measured using a digital oscilloscope (GW Instek GDS‐1074B). For short‐circuit current ($I_{\mathrm{sc}}$) measurement, a custom‐made current–voltage (I−V) converter was connected to the oscilloscope. The output power was calculated using the equation $P=V^{2}/R$. The dielectric constant of the composites was measured using an alpha frequency analyzer across a frequency range of 10^−2^ Hz to 10^6^ Hz. All TENG electrical measurements were carried out in controlled laboratory conditions (temperature ∼22°C–25°C and relative humidity ∼40%–50%) to minimize environmental variability.

### 4.5 Materials Characterization

Raman spectroscopy analysis was performed using a Witec Alpha 300R Raman microscope with a 532 nm laser. Morphological and compositional analyses were conducted using SEM (TESCAN MIRA3 XMU) with energy‐dispersive x‐ray spectroscopy (EDX) (Oxford Instruments). Additionally, XPS (Kratos AXIS SupraTM) and XRD (Rigaku SmartLab) were utilized to identify chemical states and crystalline structures of MR particles. Surface topological analysis was performed using CLSM (Olympus LEXT OLS4100), with a laser wavelength of 405 nm. Novocontrol Alpha‐A analyzer was used for dielectric measurements.

### 4.6 Measurements Consent

We obtained informed consent of human subject participating in measurements.

## Author Contributions

Shidhin Mappoli conceptualized the study, carried out composite fabrication, device fabrication, circuit design, characterizations, data analysis, and prepared the first draft of the manuscript. Keval K. Sonigara was involved in methodology, revised the original draft, and mentoring. Martin Pumera supervised the project and provided the direction. All authors contributed to the writing.

## Conflicts of Interest

The authors declare no conflicts of interest.

## Supporting information




**Supporting File 1**: smll74734‐sup‐0001‐SuppMat.pdf.

**Supporting File 2**: smll74734‐sup‐0002‐VideoS1.mp4.

**Supporting File 3**: smll74734‐sup‐0003‐VideoS2.mp4.

## Data Availability

The data that support the findings of this study are available from the corresponding author, Martin Pumera, upon reasonable request.
