# Supplementary material for: Martian‑Regolith Simulant Confined Nanogenerators for Wireless Tactile Sensing for Human‐Machine Interface
Source: Small. 2026 Aug 3;22(57):e74734. doi: 10.1002/smll.74734 (PMC13659791; doi:10.1002/smll.74734)
Supplement: Supplementary file 1 [file SMLL-22-e74734-s003.pdf]

Supporting information

Martian-regolith simulant confined nanogenerators for wireless tactile sensing for human-machine interface

Shidhin Mappoli [1], Keval K. Sonigara [1] and Martin Pumera [1,2,3,4]*

[1]Future Energy and Innovation Laboratory, Central European Institute of Technology, Brno University of Technology, Purkyňova 123, Brno 61200, Czech Republic

[2]Office of Research and Development, Asia University, Taichung 40402, Taiwan

[3]Faculty of Electrical Engineering and Computer Science, VSB - Technical University of Ostrava, 17. listopadu 2172/15, Ostrava 70800, Czech Republic

[4]Department of Medical Research, China Medical University Hospital, China Medical University, No. 91 Hsueh-Shih Road, Taichung 40402, Taiwan

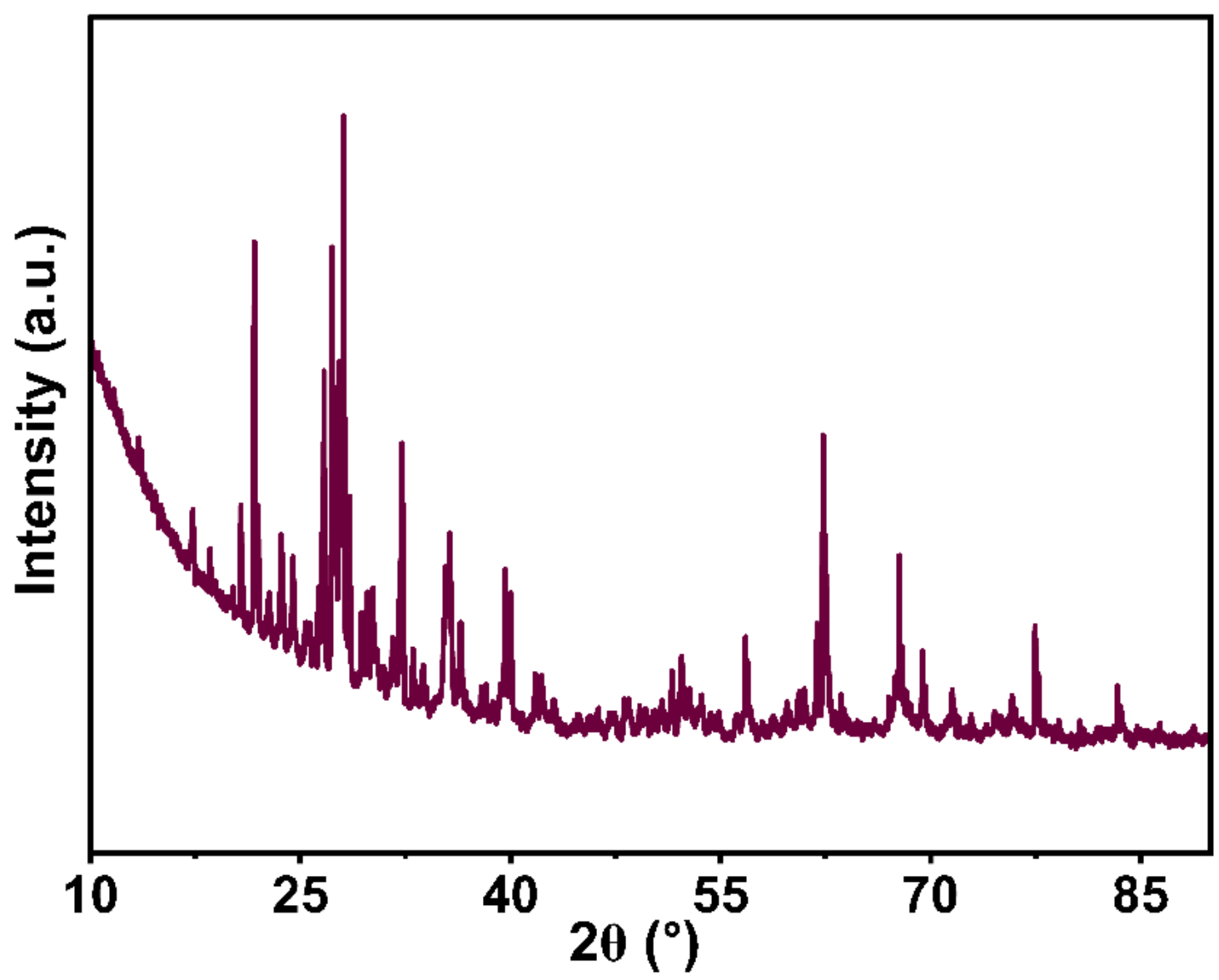

Figure S1. X-ray diffraction (XRD) pattern revealing crystalline components of MR (MGS1).

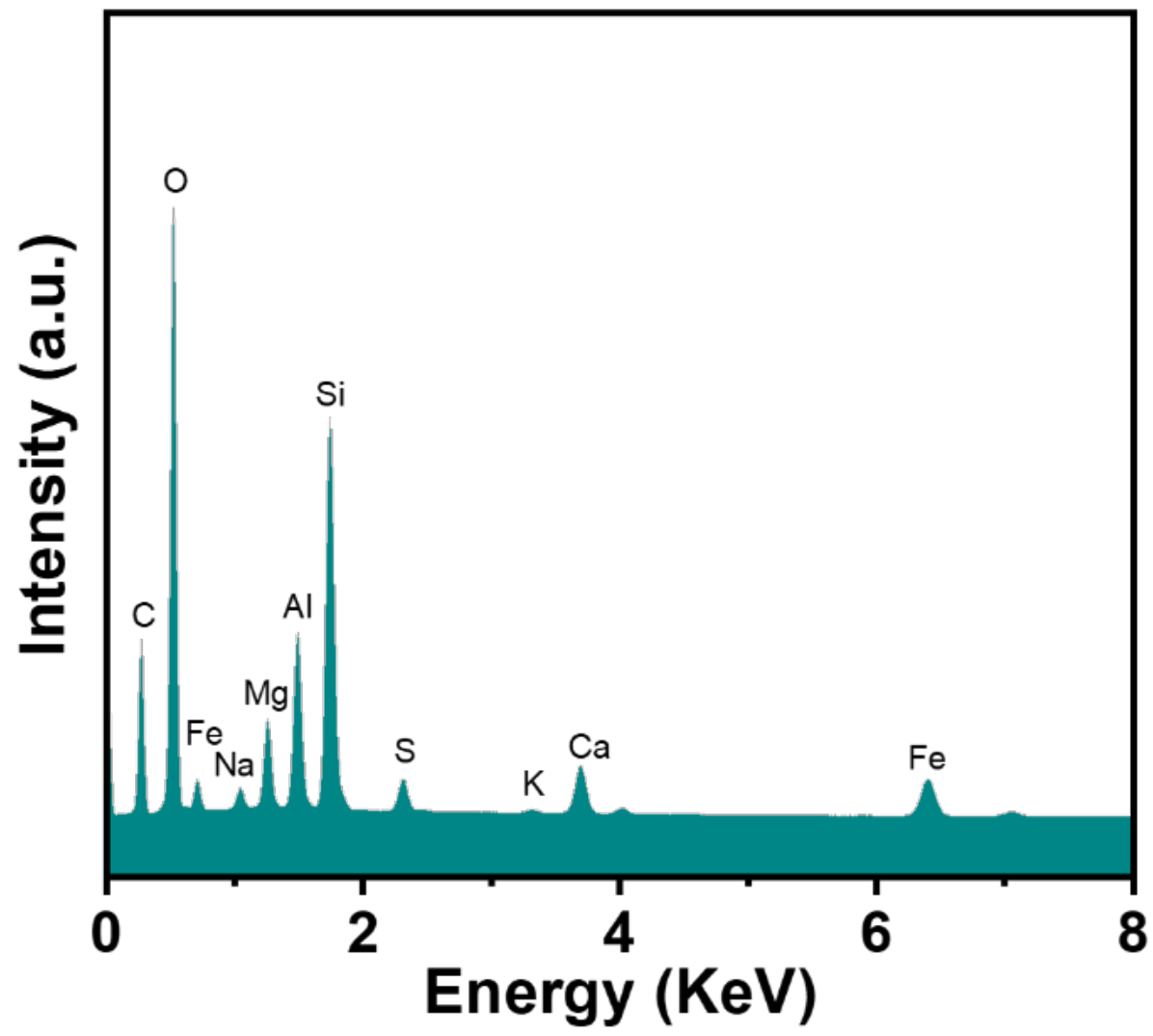

Figure S2. Energy-dispersive X-ray spectroscopy (EDX) spectrum of the MR.

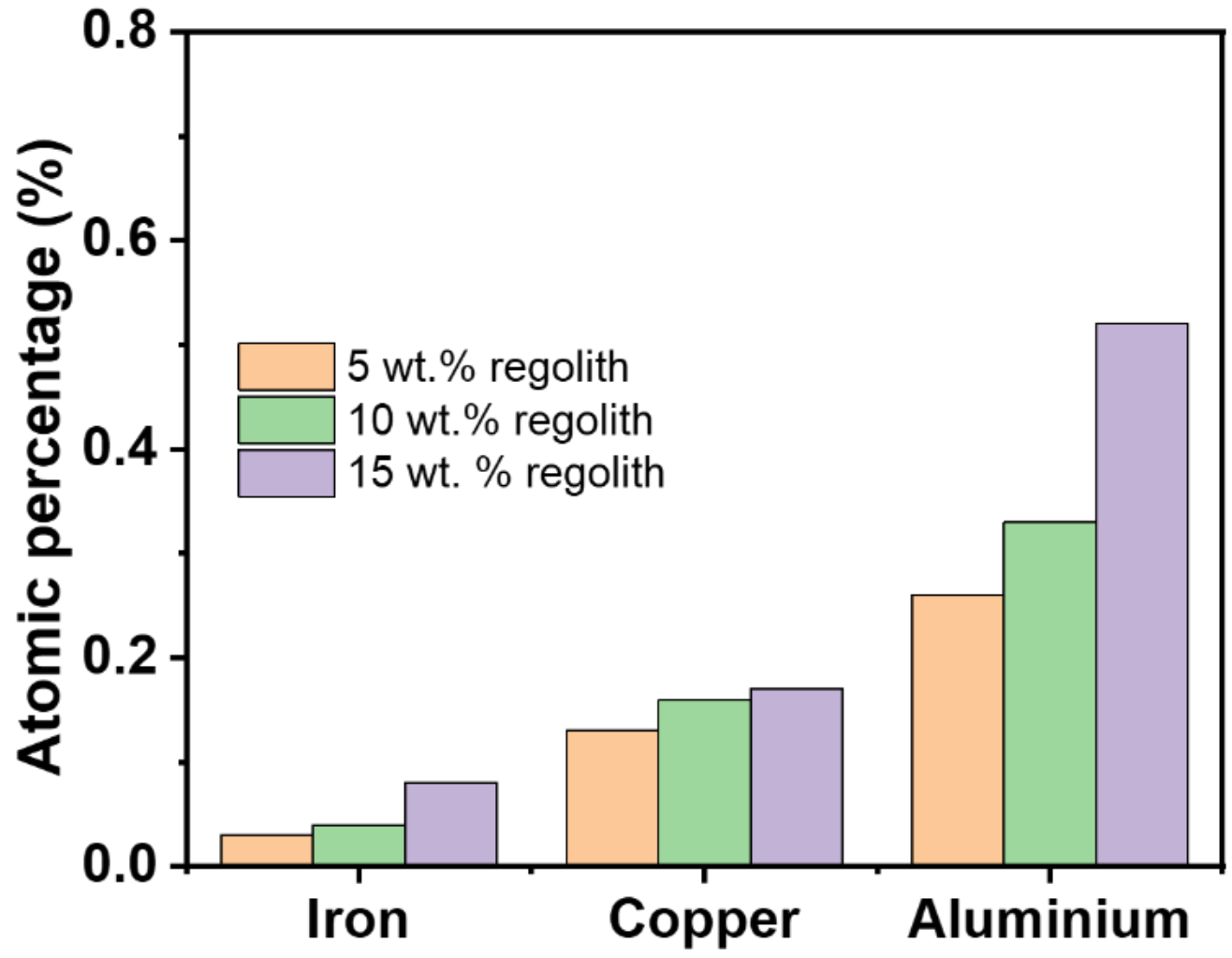

Figure S3. Elemental atomic percentages measured over a 50 µm × 50 µm area, demonstrating an increase in key elements with concentration in the scanned region.

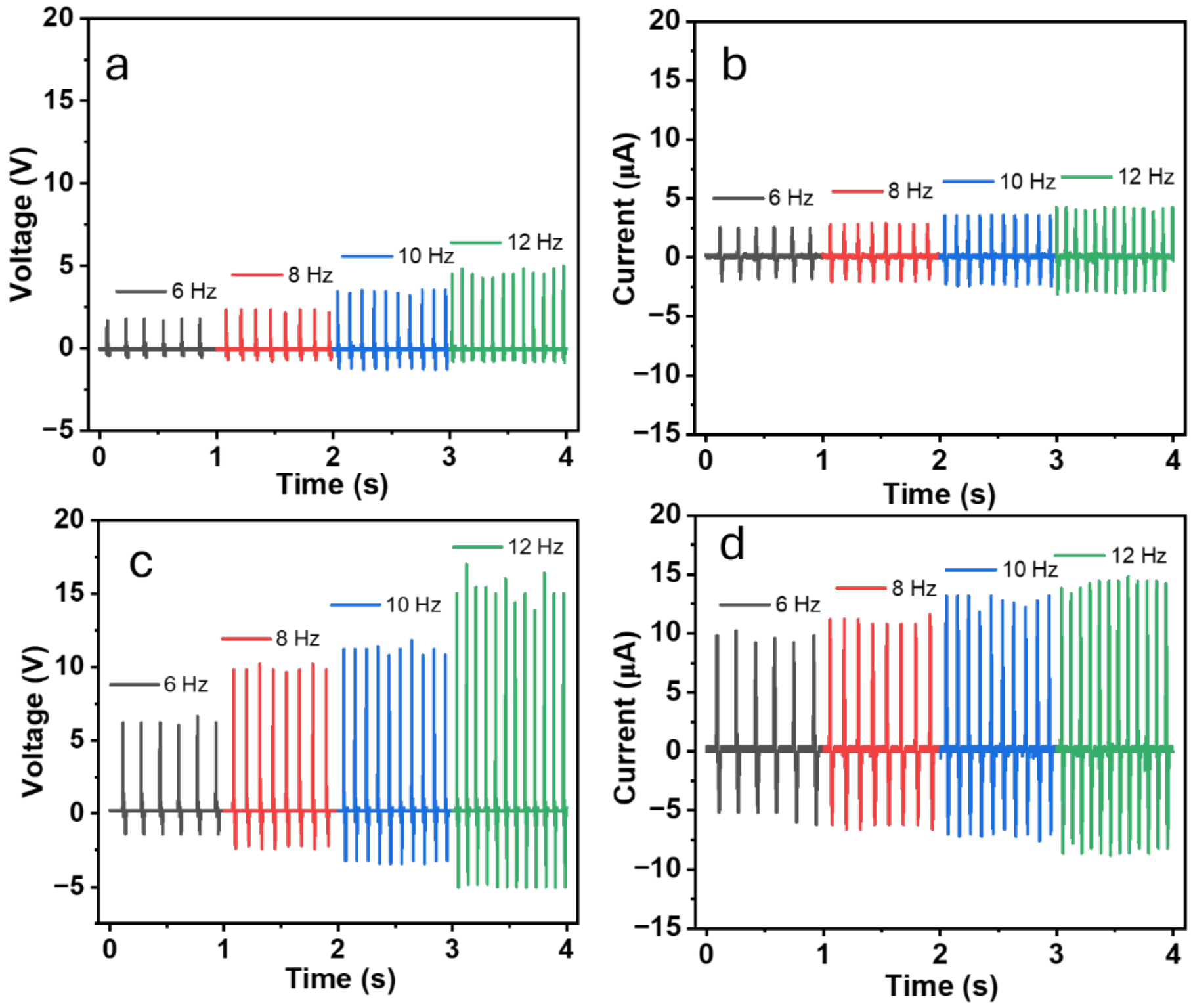

Figure S4. Electrical output of regolith–PDMS TENG with cotton counter electrode. (a) Voltage output of pure PDMS. (b) Corresponding current output of pure PDMS. (c) Voltage output of PDMS composite containing 5 wt% Martian regolith. (d) Corresponding current output of the 5 wt% regolith–PDMS composite. All measurements were conducted using a cotton fabric counter electrode to simulate soft-contact interaction.

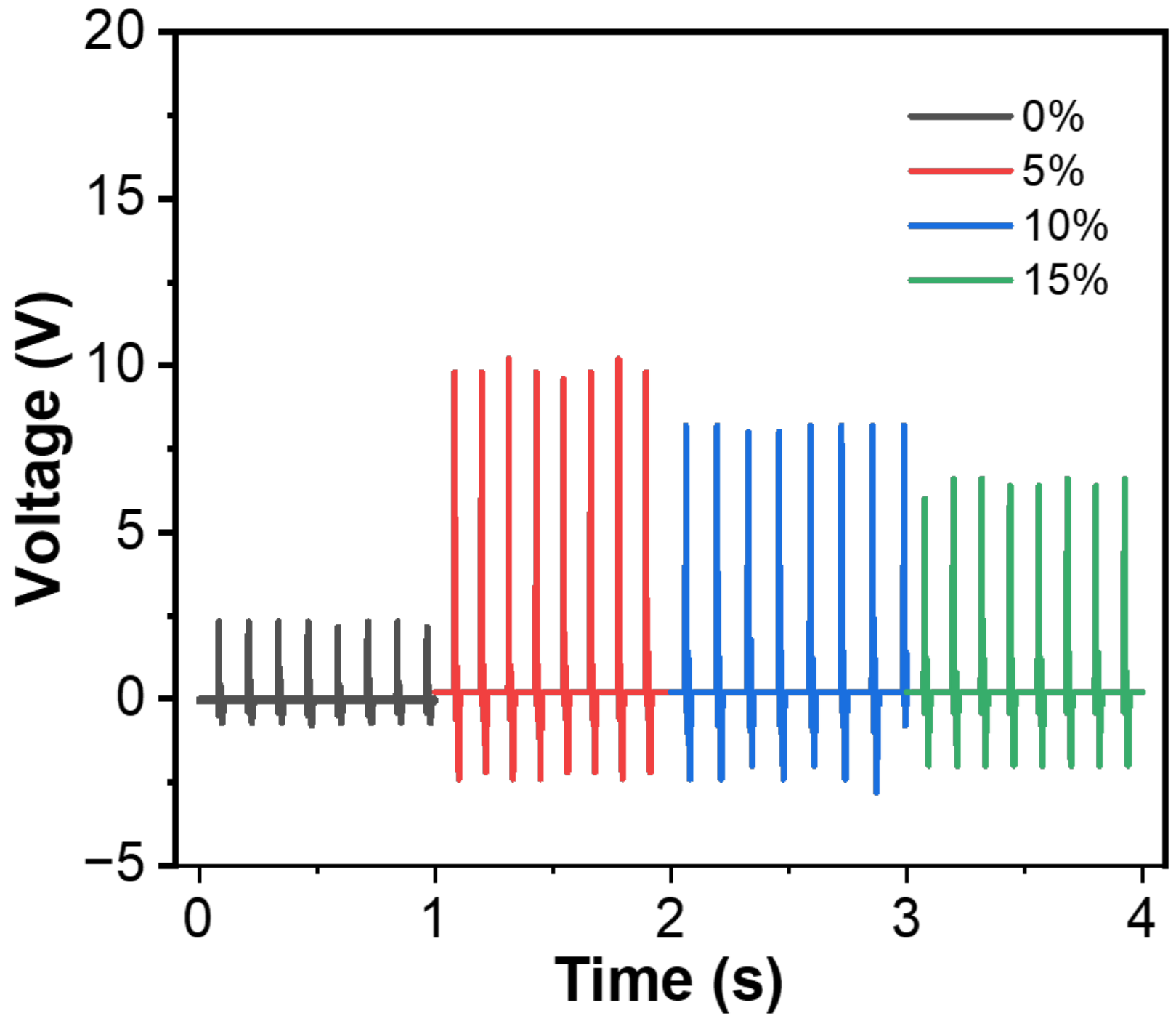

Figure S5. Voltage output comparison of MR–PDMS TENGs at varying concentrations. Voltage output of TENG devices fabricated with 0 wt%, 5 wt%, 10 wt%, and 15 wt% Martian regolith content using a cotton counter electrode.

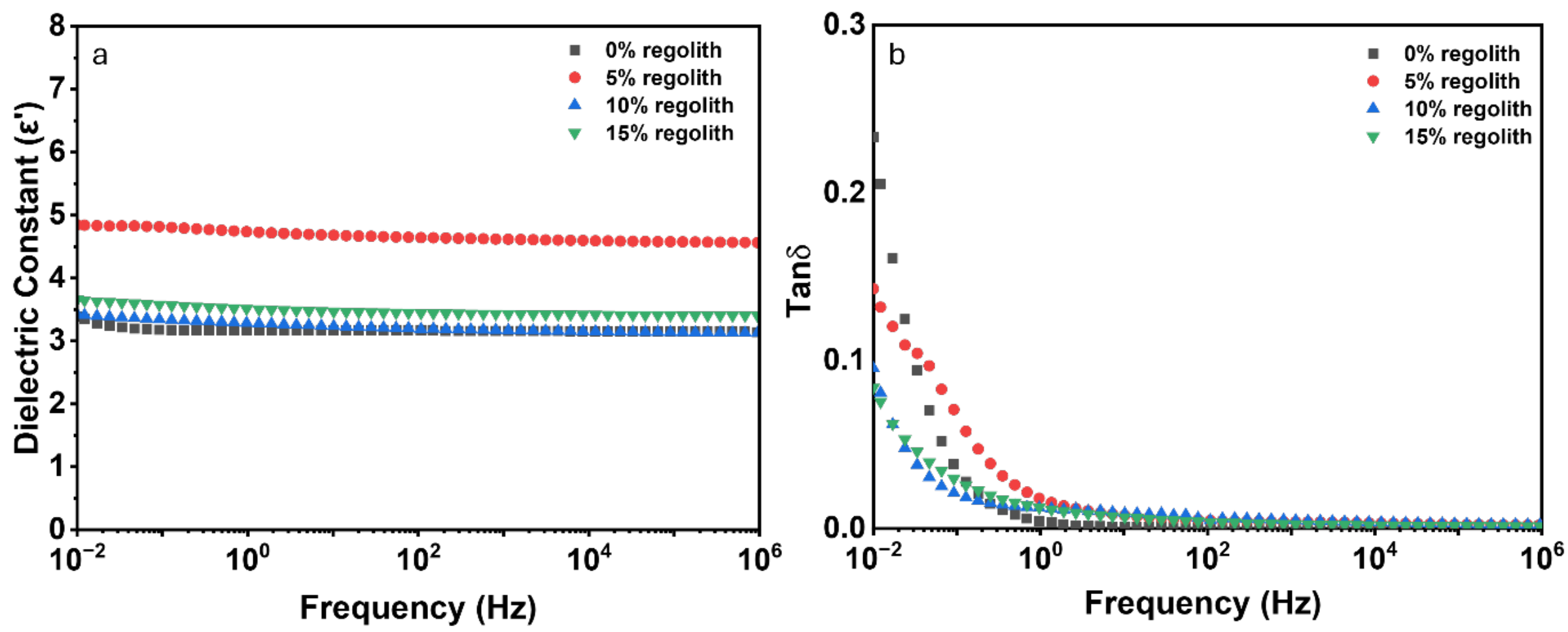

Figure S6. Dielectric properties of MR–PDMS composites. (a) Relative permittivity (ε′) of composites with varying MR concentrations, measured across a frequency range. (b) Dielectric loss tangent (tan δ) as a function of frequency, indicating energy dissipation behaviour of each composition.

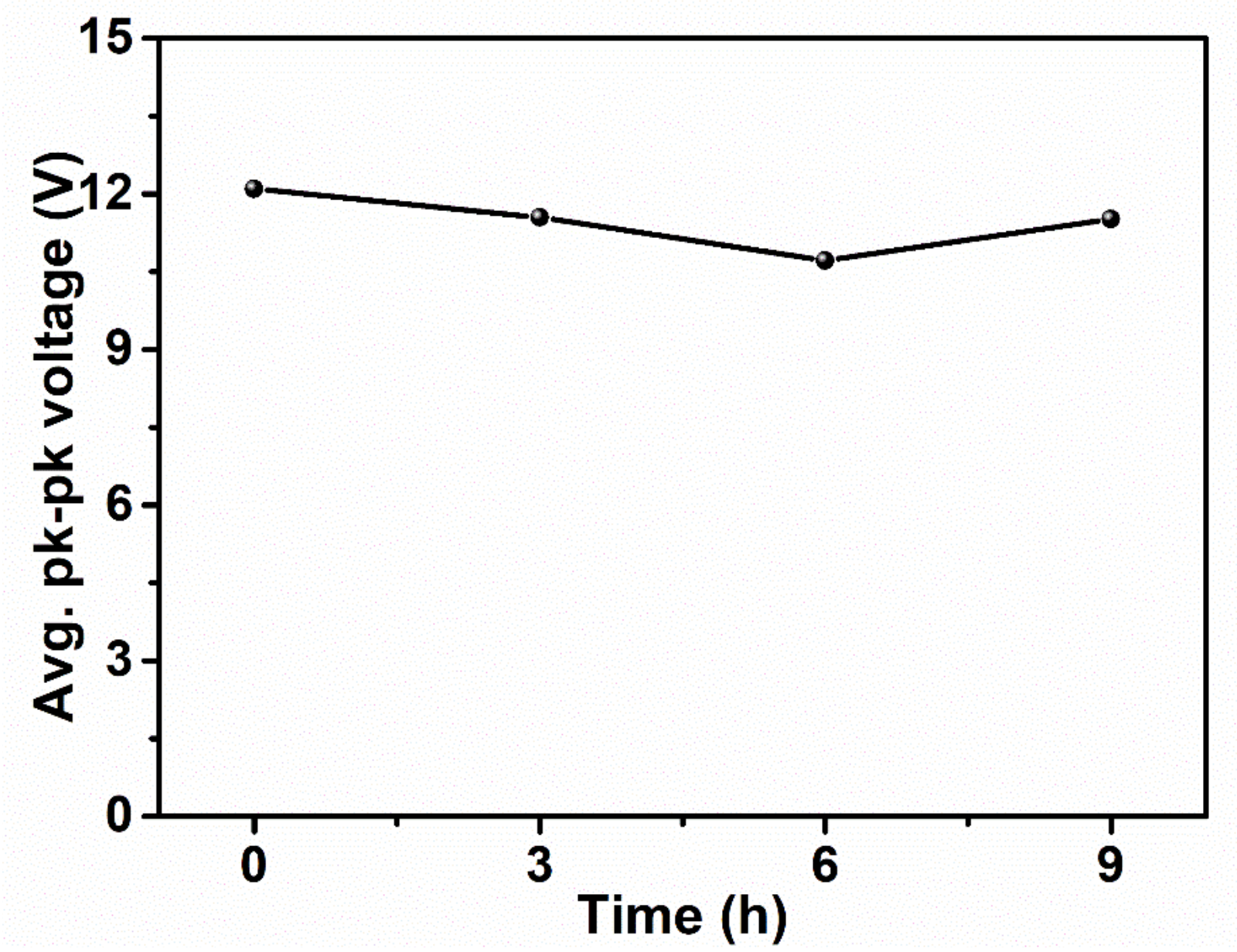

Figure S7. Long-term cyclic stability of the MR–PDMS TENG measured after different operating durations (0–9 h).

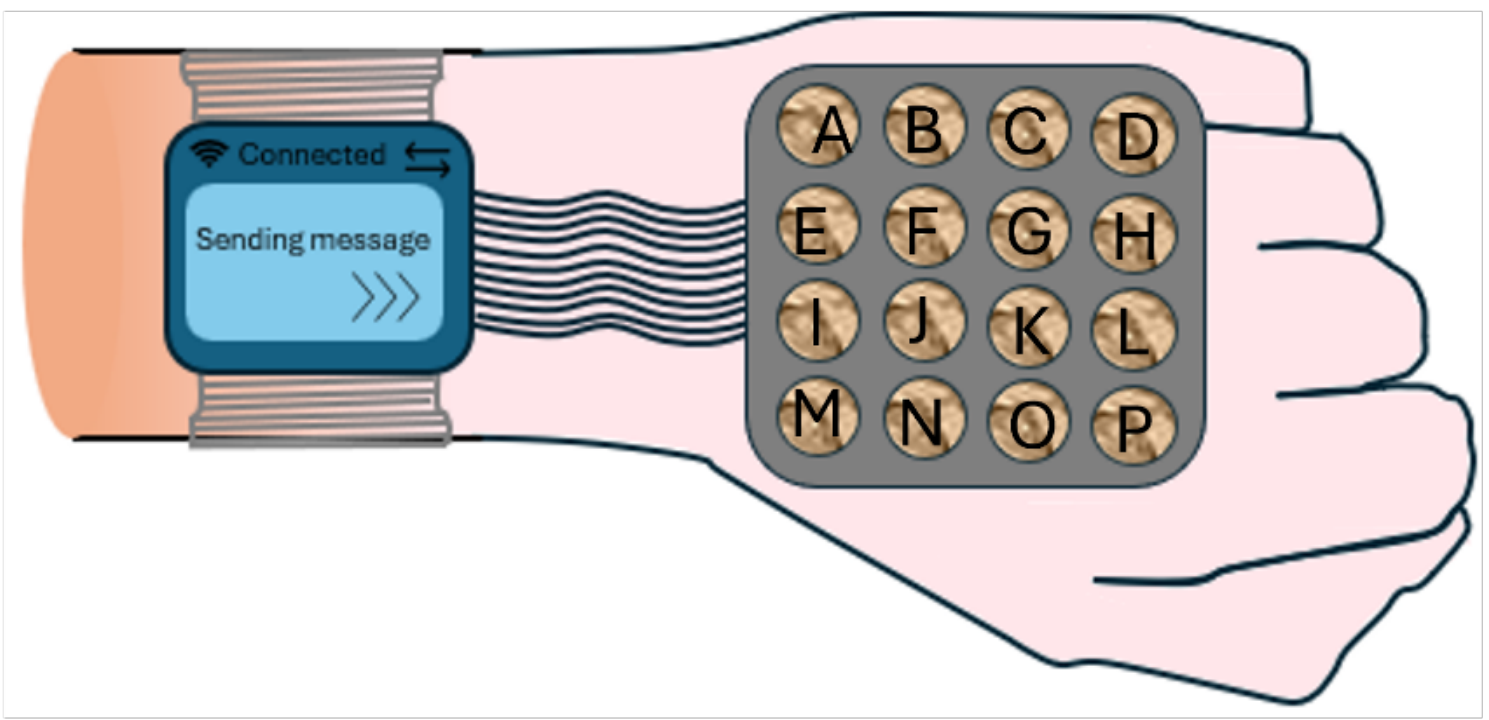

Figure S8. Schematic illustration of a keypad utilizing MR–PDMS TENGs as active sensing elements, designed for touch-based input.
